# The Association Between Body Roundness Index and Cardiovascular Disease Risk: An Analysis Based on the CHARLS Database

**DOI:** 10.31083/RCM39048

**Published:** 2025-09-23

**Authors:** Yazhao Sun, Pei Sun, Jianfeng Liu, Shiwei Cui, Yongle Li, Yuanyuan Zuo

**Affiliations:** ^1^Department of Cardiology, Cangzhou People's Hospital, 061000 Cangzhou, Hebei, China; ^2^Department of Endocrinology and Metabolism, Cangzhou People's Hospital, 061000 Cangzhou, Hebei, China

**Keywords:** body roundness index, CHARLS, association, cardiovascular disease

## Abstract

**Background:**

The body roundness index (BRI) offers a more precise evaluation of body fat and visceral fat levels. However, studies on the relationship between BRI and the risk of cardiovascular disease (CVD) remain limited.

**Methods:**

Survival differences across BRI quartiles were estimated using Kaplan-Meier analysis. The association between the BRI and the risk of CVD was examined through Cox proportional hazards and restricted cubic spline (RCS) models. Additional subgroup and sensitivity analyses were also conducted.

**Results:**

This study included 6401 patients (47.43% male), with an incidence of CVD of 17.51%. Kaplan-Meier survival analysis revealed statistically significant differences between groups based on the assigned BRI quartiles. Cox models revealed a strong association between the BRI and CVD risk, while RCS models showed a non-linear link between higher BRIs and increased CVD risk. In certain subgroups, an elevated BRI was closely correlated with an increased incidence of CVD. Notable interactions were found between BRI and gender, age, hypertension, diabetes, alcohol consumption, and smoking status. Sensitivity analysis excluding early CVD cases yielded consistent results.

**Conclusion:**

A significant non-linear association was found between the BRI and CVD risk. The BRI could be a valuable and sensitive marker for identifying individuals at high risk of CVD, with varying predictive value across different population subgroups.

## 1. Introduction

The aging population and rising prevalence of unhealthy lifestyles have increased the global burden of cardiovascular disease (CVD). Between 1990 and 2019, the number of CVD cases worldwide increased by almost two-fold, from 271 million to 523 million, while the annual number of deaths increased from 12.1 million to 18.6 million [1]. In China alone, CVD has become the major cause of death, accounting for 40% of deaths in 2010 [2].

Obesity is a major and modifiable CVD risk factor linked by the mechanisms of inflammation, insulin resistance, and abnormal lipid profiles [3]. Obesity has reached epidemic status in China and globally, posing serious public health and economic challenges [4]. The traditional marker for overall obesity is body mass index (BMI) [5, 6], although this does not consider fat distribution and variability in body composition, with the latter being perhaps more important for CVD risk estimation. A study of 36,656 subjects by Lee *et al*. [7] revealed the visceral-to-subcutaneous fat ratio was more closely associated with all-cause mortality than general obesity, as indicated by BMI. Other studies have also reported that abdominal fat distribution was a stronger predictor for CVD than general obesity [8, 9].

Thomas *et al*. [10] introduced the body roundness index (BRI) in 2013. This novel anthropometric index combines height and waist circumference measurements to estimate both visceral and overall body fat using mathematical modeling. Various studies have since demonstrated significant correlations between BRI and several metabolic conditions, including diabetes, hypertension, hyperuricemia, and metabolic syndrome, with several of the associations even surpassing those of conventional anthropometric measurements [11, 12, 13, 14]. A recent large-scale, cross-sectional analysis of 17,360 participants from a Chinese population confirmed significant correlations between BRI and a composite of cardiometabolic risk factors [15]. Despite the promising preliminary evidence of BRI as a possible indicator of cardiometabolic health, there is currently only limited longitudinal evidence on its relation with CVD risk in representative national samples. In addition, the dose-response relationship between BRI and CVD risk, as well as its stability in diverse subgroups, has yet to be explored. Using data from the China Health and Retirement Longitudinal Study (CHARLS), the aim of this analysis was to explore the relationship between BRI and CVD risk, examine how this relationship varies across demographic and clinical subgroups, and investigate potential non-linear dose-response patterns between BRI and CVD risk.

## 2. Methods

### 2.1 Study Participants

The dataset for this analysis was obtained from the CHARLS database. CHARLS began in 2011 with support and funding from Peking University to develop a nationally representative and interdisciplinary longitudinal survey. At baseline (2011–2012), the study recruited 16,931 subjects aged 45 years and older. Those with missing BRI, blood lipid, blood glucose, and other pertinent information were excluded, together with those who had a physician-diagnosed CVD (e.g., cardiac event or stroke) or were lost to follow-up (n = 10,530). A total of 6401 subjects were included in the final analysis of the association between BRI and CVD incidence. Participants were divided into quartiles according to the distribution of BRI in the study population (Q1, Q2, Q3, and Q4). The cut-off points for these quartiles were set by the 25th, 50th, and 75th percentiles of the baseline BRI distribution, thus creating relatively balanced group sizes for evaluation of possible nonlinear associations with CVD risk. The CHARLS study was approved by the Institutional Review Board of Peking University, and all subjects provided written informed consent. All of the data are publicly accessible for research purposes on the CHARLS website (http://charls.pku.edu.cn/en).

### 2.2 Data Collection and Definition

The demographic data collected included the variables of gender, age, BMI, marital status (“married” or “other”), residential setting (“urban” or “rural”), waist circumference, alcohol consumption (“never” or “current/past”), and smoking status (“never” or “current/past”). Information on comorbidities, including hypertension and diabetes, was also collected.

Laboratory tests recorded data on various biomarkers including white blood cell (WBC) count, platelet (PLT) count, and the levels of hemoglobin (HGB), high-sensitivity C-reactive protein (hsCRP), hemoglobin A1c (HbA1c), glucose (GLU), serum uric acid (SUA), blood urea nitrogen (BUN), serum creatinine (Scr), triglycerides (TG), total cholesterol (TC), high-density lipoprotein cholesterol (HDL-C), and low-density lipoprotein cholesterol (LDL-C).

The BRI was computed using the following formula, as described previously [10]:

BRI = 364.2 – 365.5 × (1 – [waist circumference (m)/2π]^2^/[0.5 × height(m)]^2^)^½^

### 2.3 Study Outcomes

Participants were interviewed face-to-face every two years by computer-assisted personal interviews. The study outcomes were CVD incidence, i.e., cardiac events or stroke. CVD was assessed by questions such as: “Has a physician ever informed you that you have had a heart attack, angina, coronary heart disease, heart failure, or any heart problems?”, or “Has a physician ever informed you that you have had a stroke?”. Persons with heart diseases or stroke were coded as having CVD. Follow-ups were carried out in 2013–2014 (Wave 2), 2015–2016 (Wave 3), 2017–2018 (Wave 4), and 2019–2020 (Wave 5).

### 2.4 Statistical Analysis

The baseline characteristics of participants are described as percentages for categorical variables, as mean with standard deviation (mean ± SD) for normally distributed variables, and as median with interquartile ranges (median [IQR]) for non-normally distributed variables. Analysis of variance, Kruskal-Wallis H test, and chi-square tests were used to compare baseline characteristics between different BRI groups. Kaplan-Meier analysis was applied to estimate the incidence of outcome events in different groups stratified according to BRI, with the log-rank test used to identify significant differences.

Cox regression models were used to assess the relationship between BRI and CVD, with hazard ratios (HRs) and 95% confidence intervals (CIs) calculated for the incidence of CVD. BRI was evaluated in two ways: as a continuous variable and as a categorical variable, with the lowest quartile (Q1) used as the reference category for the latter. Model 1 was a crude model with no adjustments. Model 2 included adjustments for gender and age. Model 3 included further adjustments of Model 2 for BMI, marital status, residential setting, alcohol consumption, smoking status, hypertension, and diabetes, as well as the laboratory variables of WBC, HGB, PLT, hsCRP, HbA1c, GLU, BUN, SUA, Scr, TG, TC, LDL-C, and HDL-C. To control the effect of multicollinearity on model stability, variance inflation factors (VIFs) were estimated before the construction of Model 3. Only those variables with a VIF <10 were included in the final analysis model.

Restricted cubic spline (RCS) analysis was also performed to explore the dose-response relationship between BRI and CVD. To further assess the relationship between BRI and CVD risk, subgroup analyses were conducted using multivariable Cox regression models stratified by gender, age (<60 years and ≥60 years), marital status, residential setting, alcohol consumption, smoking status, hypertension, and diabetes. A sensitivity analysis was conducted to assess the robustness of the relationship between BRI and CVD incidence. All statistical analyses in the present study were performed using the R Programming Language 4.4.1 (R Foundation for Statistical Computing, Vienna, Austria), with statistical significance defined as a two-sided *p*-value of <0.05.

## 3. Results

### 3.1 Baseline Traits

Table 1 presents the baseline clinical characteristics of patients categorized according to BRI quartiles: Q1 (<3.12), Q2 (3.12–3.96), Q3 (3.96–4.93), and Q4 (>4.93). Significantly higher percentages of current/past smoking status, hypertension and diabetes were found in the Q4 group, as well as increased waist circumference, BMI, WBC, HbA1c, GLU, SUA, Scr, LDL-C, TC, TG, and hsCRP. The Q4 group had a lower percentage of married and rural residents, and lower HGB and HDL-C levels. The prevalence of CVD increased progressively with increasing BRI quartiles (6.50% vs. 15.04% vs. 23.01% vs. 25.50%, *p* < 0.001).

**Table 1.** **Baseline characteristics**.

Category		Overall (N = 6401)	Q1 (N = 1600)	Q2 (N = 1602)	Q3 (N = 1599)	Q4 (N = 1600)	*p*-value
Demographic							
	Age, years, mean ± SD	58.88 ± 9.52	58.71 ± 9.35	59.43 ± 9.68	58.72 ± 9.59	58.66 ± 9.44	0.058
	Male, n (%)	3036 (47.43%)	760 (47.50%)	779 (48.63%)	722 (45.15%)	775 (48.44%)	0.179
	Marital status (married), n (%)	5618 (87.77%)	1401 (87.56%)	1420 (88.64%)	1444 (90.31%)	1353 (84.56%)	<0.001
	Residential setting (rural), n (%)	4078 (63.71%)	1099 (68.69%)	1079 (67.35%)	979 (61.23%)	921 (57.56%)	<0.001
	Waist circumference, m, median (IQR)	0.84 (0.77–0.91)	0.73 (0.70–0.77)	0.81 (0.77–0.84)	0.87 (0.84–0.91)	0.95 (0.91–1.00)	<0.001
	BMI, kg/m^2^, mean ± SD	23.20 ± 3.82	19.84 ± 2.29	21.94 ± 2.47	24.10 ± 2.77	26.92 ± 3.47	<0.001
	Alcohol consumption (current/past), n (%)	2463 (38.48%)	595 (37.19%)	639 (39.89%)	594 (37.15%)	635 (39.69%)	0.199
	Smoking status (current/past), n (%)	2500 (39.06%)	537 (33.56%)	635 (39.64%)	601 (37.59%)	727 (45.44%)	<0.001
Comorbidity							
	Hypertension, n (%)	1322 (20.65%)	160 (10.00%)	252 (15.73%)	353 (22.08%)	557 (34.81%)	<0.001
	Diabetes, n (%)	302 (4.72%)	29 (1.81%)	59 (3.68%)	85 (5.32%)	129 (8.06%)	<0.001
Laboratory tests							
	WBC, K/uL, median (IQR)	5.90 (4.90–7.20)	5.90 (4.90–7.00)	5.90 (4.90–7.17)	5.90 (4.90–7.20)	5.90 (4.94–7.30)	0.009
	PLT, K/uL, median (IQR)	205.00 (162.00–254.00)	201.00 (159.00–250.00)	208.00 (164.00–260.75)	208.00 (165.50–254.50)	206.00 (159.00–255.00)	0.297
	HGB, g/dL, median (IQR)	14.30 (13.10–15.50)	14.50 (13.30–15.70)	14.60 (13.40–15.80)	14.10 (13.00–15.30)	13.90 (12.70–15.20)	<0.001
	HbA1c, %, mean (SD)	5.22 (0.77)	5.18 (0.49)	5.20 (0.65)	5.23 (0.81)	5.27 (1.03)	0.007
	GLU, mg/dL, mean (SD)	109.66 (33.82)	108.29 (24.20)	108.17 (27.83)	109.51 (32.71)	112.66 (46.19)	<0.001
	BUN, mg/dL, median (IQR)	15.04 (12.55–18.01)	14.94 (12.44–17.65)	15.10 (12.63–18.29)	15.10 (12.62–18.02)	15.07 (12.55–18.19)	0.268
	SUA, mg/dL, median (IQR)	4.27 (3.54–5.10)	4.20 (3.50–5.02)	4.24 (3.52–5.05)	4.32 (3.56–5.16)	4.34 (3.58–5.20)	<0.001
	Scr, mg/dL, median (IQR)	0.76 (0.64–0.88)	0.75 (0.64–0.87)	0.75 (0.64–0.87)	0.76 (0.64–0.88)	0.76 (0.64–0.89)	0.013
	HDL-C, mmol/L, mean (SD)	1.31 (0.39)	1.34 (0.40)	1.34 (0.40)	1.30 (0.40)	1.28 (0.36)	<0.001
	TG, mmol/L, median (IQR)	1.20 (0.85–1.76)	1.14 (0.83–1.72)	1.17 (0.83–1.69)	1.23 (0.88–1.78)	1.26 (0.88–1.83)	<0.001
	LDL-C, mmol/L, mean (SD)	2.99 (0.90)	2.96 (0.82)	2.93 (0.91)	3.03 (0.89)	3.05 (0.94)	<0.001
	TC, mmol/L, mean (SD)	4.96 (0.97)	4.91 (0.89)	4.94 (0.97)	4.96 (0.98)	5.02 (1.04)	0.017
	hsCRP, mg/L, median (IQR)	0.98 (0.54–2.05)	0.94 (0.52–1.95)	0.89 (0.50–1.85)	1.06 (0.55–2.17)	1.04 (0.58–2.27)	<0.001
	CVD, n (%)	1121 (17.51%)	104 (6.50%)	241 (15.04%)	368 (23.01%)	408 (25.50%)	<0.001

SD, standard deviation; IQR, interquartile ranges; WBC, white blood cell; PLT, platelet; BMI, body mass index; HGB, hemoglobin; HbA1c, hemoglobin A1c; GLU, glucose; BUN, blood urea nitrogen; SUA, serum uric acid; Scr, serum creatinine; HDL-C, high-density lipoprotein cholesterol; TG, triglycerides; LDL-C, low-density lipoprotein cholesterol; TC, total cholesterol; hsCRP, high-sensitivity C-reactive protein; CVD, cardiovascular disease.

### 3.2 Association Between BRI and CVD

**Fig. 1.**
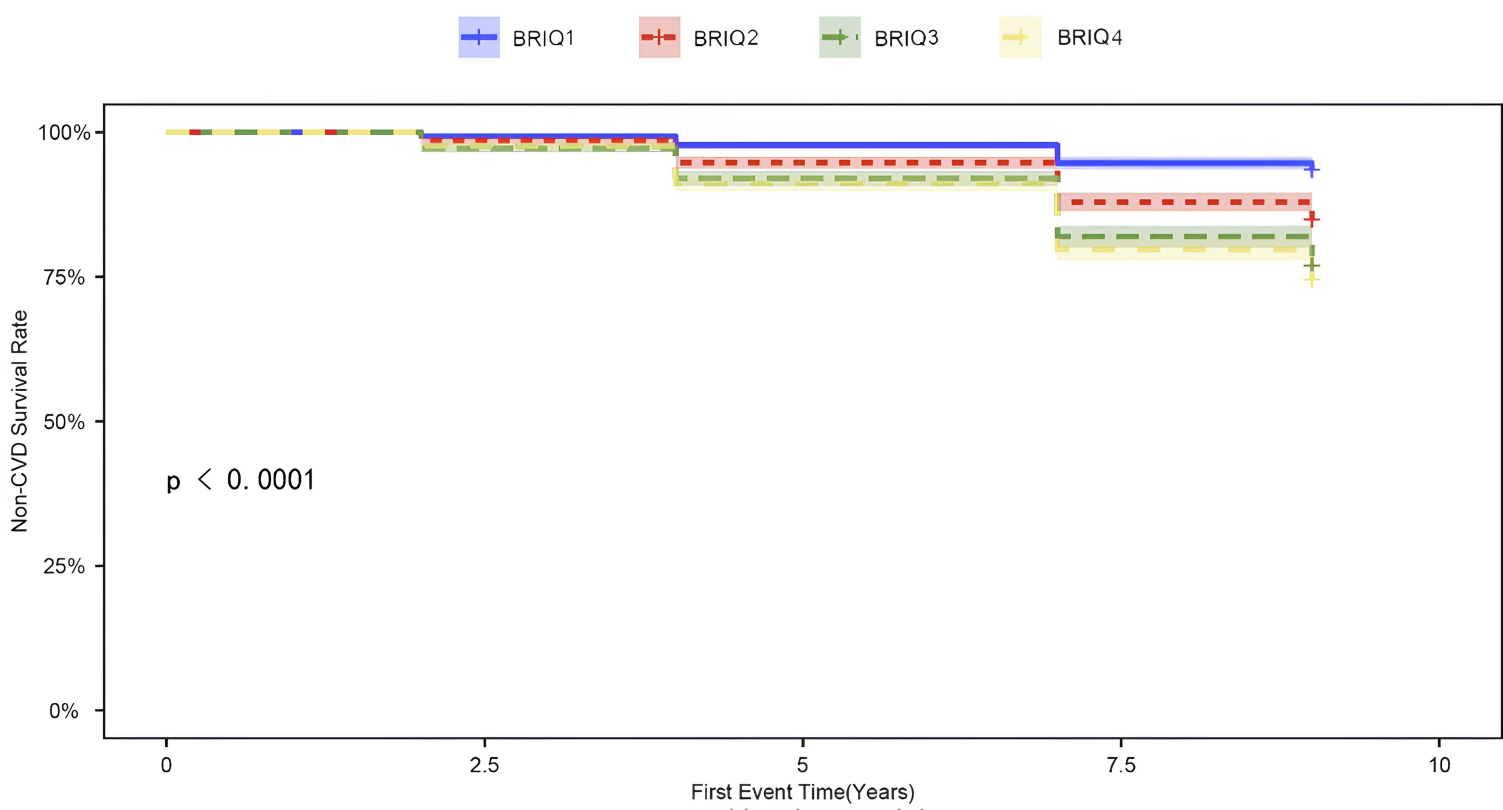
**Cumulative incidence curve of non-CVD events**. CVD, cardiovascular disease; BRI, body roundness index.

Fig. 1 displays the cumulative incidence curves for non-CVD events according to BRI quartiles. Statistically significant differences in the incidence of non-CVD events between the groups were observed throughout the follow-up period (*p* < 0.0001). These results indicate the occurrence of non-CVD events was notably different between the BRI groups, further underscoring the relevance of BRI as a potential risk factor.

When BRI was treated as a continuous measure, Cox regression analysis revealed a significant association between BRI and CVD risk across different models. In the fully adjusted model, the HR was slightly lower at 1.33 (95% CI 1.25–1.40, *p* < 0.001). Each unit increase in BRI was associated with an increase of approximately 33% in CVD risk. These results highlight the strong, positive relationship between BRI and CVD risk, and suggest that BRI could serve as a valuable early screening tool for identifying individuals at risk of CVD, thus potentially favoring timely intervention to reduce the incidence. Additionally, when BRI was categorized into quartiles, significant associations with CVD risk were observed across the higher quartiles in the fully adjusted model with Q1 as the baseline: for Q2, HR = 2.12 (95% CI 1.72–2.75, *p* < 0.001); for Q3, HR = 2.99 (95% CI 2.33–3.83, *p* < 0.001); and for Q4, HR = 3.14 (95% CI 2.50–3.96, *p* < 0.001) (see Table 2).

**Table 2.** **BRI impact on CVD risk**.

Categories		Model 1		Model 2		Model 3	
BRI		HR (95% CI)	*p*-value	HR (95% CI)	*p*-value	HR (95% CI)	*p*-value
Continuous		1.36 (1.32–1.40)	<0.001	1.36 (1.32–1.40)	<0.001	1.33 (1.25–1.40)	<0.001
Categories							
	Q1 (N = 1600)	Reference	1.000	Reference	1.000	Reference	1.000
	Q2 (N = 1602)	2.43 (1.93–3.05)	<0.001	2.41 (1.91–3.03)	<0.001	2.12 (1.72–2.75)	<0.001
	Q3 (N = 1599)	3.88 (3.12–4.83)	<0.001	3.88 (3.12–4.82)	<0.001	2.99 (2.33–3.83)	<0.001
	Q4 (N = 1600)	4.37 (3.52–5.42)	<0.001	4.37 (3.52–5.42)	<0.001	3.14 (2.50–3.96)	<0.001
*p* for trend			<0.001		<0.001		<0.001

Model 1: Unadjusted; Model 2: Adjusted for gender and age; Model 3: Further adjusted for BMI, marital status, residential setting, alcohol consumption, smoking status, hypertension, diabetes, and various lab markers (WBC, PLT, HGB, HbA1c, GLU, BUN, SUA, Scr, HDL-C, TG, LDL-C, TC, hsCRP).

**Fig. 2.**
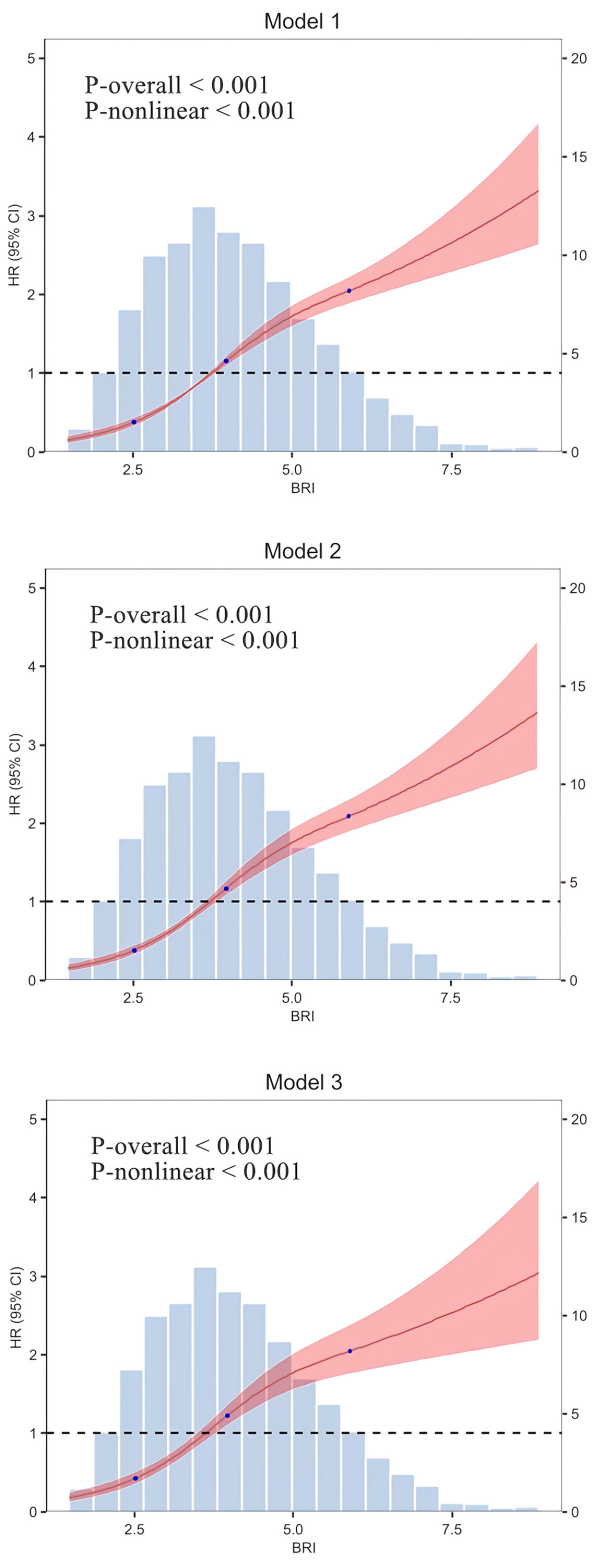
**RCS regression model**. Model 1: Unadjusted; Model 2: Adjusted for gender and age; Model 3: Further adjusted for BMI, marital status, residential setting, alcohol consumption, smoking status, hypertension, diabetes, and various lab markers (WBC, PLT, HGB, HbA1c, GLU, BUN, SUA, Scr, HDL-C, TG, LDL-C, TC, hsCRP).

The results of the RCS regression model provide additional evidence of a non-linear relationship between BRI and CVD risk (Fig. 2). This analysis revealed a significant non-linear pattern in all models, whether unadjusted, partially adjusted, or fully adjusted (all non-linearity *p*-values < 0.001). This indicates the impact of BRI on CVD risk does not increase in a linear fashion, but rather accelerates significantly once certain thresholds are reached.

### 3.3 Subgroup Analysis

Additionally, we conducted risk stratification analysis of BRI across multiple subgroups according to gender, age, marital status, rural, drinking status, smoking status, hypertension, and diabetes.

Table 3 presents the HRs for these various subgroups. In all subgroups examined, BRI was significantly associated with an increased risk of CVD (all *p* < 0.05), except for the the “yes” group in Diabetes subgroup, where no significant association was found. The subgroup analyses also revealed notable interactions between BRI and the factors of gender, age, diabetes, hypertension, alcohol consumption, and smoking status (all interaction *p*-values < 0.05).

**Table 3.** **Subgroup analysis**.

Subgroup		BRI		*p* for interaction
		HR (95% CI)	*p*-value	
Gender				
	Female	1.25 (1.16–1.35)	<0.001	<0.001
	Male	1.54 (1.39–1.70)	<0.001	
Age, years				
	≥60	1.37 (1.26–1.49)	<0.001	0.015
	<60	1.32 (1.22–1.43)	<0.001	
Marital status				
	Married	1.33 (1.25–1.42)	<0.001	0.743
	Others	1.27 (1.10–1.47)	0.001	
Diabetes				
	Yes	1.23 (0.90–1.69)	0.202	<0.001
	No	1.39 (1.31–1.47)	<0.001	
Hypertension				
	Yes	1.27 (1.15–1.41)	<0.001	<0.001
	No	1.41 (1.31–1.52)	<0.001	
Alcohol consumption				
	Current/past	1.58 (1.41–1.76)	<0.001	<0.001
	Never	1.26 (1.18–1.36)	<0.001	
Smoking status				
	Current/past	1.49 (1.34–1.65)	<0.001	<0.001
	Never	1.28 (1.19–1.37)	<0.001	
Residential setting				
	Rural	1.34 (1.24–1.45)	<0.001	0.060
	Urban	1.35 (1.23–1.48)	<0.001	

Adjusted for gender, age, BMI, marital status, residential setting, alcohol consumption, smoking status, hypertension, diabetes, and various lab markers (WBC, PLT, HGB, HbA1c, GLU, BUN, SUA, Scr, HDL-C, TG, LDL-C, TC, hsCRP).

### 3.4 Sensitivity Analysis

Sensitivity analysis was performed by excluding participants with an onset of CVD within the first two years of follow-up. The findings were in agreement with the primary analysis, with no differences observed (Table 4).

**Table 4.** **Sensitivity analysis**.

BRI		HR (95% CI)	*p*-value
Continuous		1.33 (1.25–1.42)	<0.001
Categories			
	Q1 (N = 1600)	Reference	1.00
	Q2 (N = 1602)	2.20 (1.72–2.82)	<0.001
	Q3 (N = 1599)	3.04 (2.34–3.95)	<0.001
	Q4 (N = 1600)	3.08 (2.41–3.93)	<0.001
*p* for trend			<0.001

Adjusted for gender, age, BMI, marital status, residential setting, alcohol consumption, smoking status, hypertension, diabetes, and various lab markers (WBC, PLT, HGB, HbA1c, GLU, BUN, SUA, Scr, HDL-C, TG, LDL-C, TC, hsCRP).

## 4. Discussion

This analysis examined the association between BRI and CVD risk. A total of 1121 patients developed CVD over the course of the study. Kaplan-Meier analysis found significant inter-group differences according to BRI quartiles. This result strengthens the potential application of BRI as a simple and accessible tool for clinical CVD risk stratification, allowing clinicians to identify individuals who are at increased risk according to abdominal fat distribution. Adjusted Cox proportional hazards analysis revealed a significant association between BRI and CVD risk when BRI was modeled as a continuous variable (HR = 1.33, 95% CI 1.25–1.40, *p* < 0.001). When BRI was modeled as a categorical variable, the HRs for CVD in the second, third and fourth BRI groups compared to the Q1 reference group were 2.12 (95% CI 1.72–2.75), 2.99 (95% CI 2.33–3.83) and 3.14 (95% CI 2.50–3.96) respectively. These findings demonstrate the utility of BRI as an efficient tool for the discrimination of individuals with different CVD risks, further emphasizing its utility in clinical applications for risk prediction. RCS models also demonstrated a non-linear association between elevated BRI and increased CVD risk. Moreover, in certain subgroups, elevated BRI was associated with a significantly higher incidence of CVD. Significant interactions were observed between BRI and gender, age, hypertension, diabetes, alcohol consumption, smoking status, and BMI. The ability of BRI to predict CVD risk in different subgroups further supports its utility in clinical practice. Additionally, its reliability and non-invasive nature provides a useful tool for identifying high-risk individuals in resource-limited environments where costly imaging is not feasible. However, BRI may be an indicator of underlying common risk factors, such as chronic inflammation, insulin resistance, and metabolic dysfunction, rather than being a direct causative agent of CVD. This is an important consideration when interpreting the predictive function of BRI, and emphasizes the need for further research to elucidate the mechanistic processes involved.

Obesity is a chronic metabolic disorder characterized by abnormal fat deposition and metabolic derangement. It is closely associated with several diseases, including CVD, diabetes, cancer, and metabolic syndrome [16, 17]. Although the pathophysiological basis for the link between visceral fat deposition and CVD risk is well-established, the current research highlights the utility of BRI as a simple, accessible, and non-invasive tool for estimating this risk. Compared to the expensive imaging methods of computed tomography (CT) and MRI, BRI is a cost-effective option for the early determination of high cardiovascular risk in clinical practice. BRI is a new anthropometric index for measuring abdominal fat deposition. It is assessed by measuring waist circumference and height, allowing more accurate assessment of visceral fat and body fat distribution. Excess deposition of visceral fat is a well-known and potent risk factor for CVD, with elevated BRI being associated with metabolic disturbances such as insulin resistance, diabetes, hypertension, dyslipidemia, and metabolic syndrome [18, 19, 20, 21, 22, 23]. For example, a prospective cohort study conducted in Southwest China and involving 9280 participants found that for each unit increase in BRI, the risk of hypertension increased by 17% after adjusting for confounding factors [11]. A study by Cai *et al*. [24] on patients with hypertension and obstructive sleep apnea also validated the predictive ability of BRI for CVD risk. A large cross-sectional study conducted by Li *et al*. [25] showed that BRI outperforms other indices in predicting CVD risk in the Chinese population.

In the present analysis, a positive correlation was found between increased BRI and CVD risk after controlling for appropriate covariates. The main strength of our study was its detailed exploration of how different covariates affect the association between BRI and CVD risk, further supporting the utility of BRI as an assessment tool for cardiovascular risk. Our results add to the clinical evidence of an association between BRI and CVD risk, emphasizing the potential utility of BRI as a simple yet accessible marker for the screening of high-risk groups. Several studies have demonstrated that elevated BRI is significantly associated with CVD, cardiovascular-related deaths, and overall mortality [26, 27, 28, 29]. Although the current study did not directly investigate the specific biological mechanisms involved, existing research suggests that excessive accumulation of abdominal fat may increase the CVD risk through inflammatory responses and metabolic disturbances. Previous studies have shown that abdominal fat accumulation may lead to chronic low-grade inflammation, thereby affecting insulin sensitivity, blood pressure regulation, and lipid metabolism, all of which increase the risk of CVD [30, 31, 32, 33, 34, 35, 36]. Furthermore, the accumulation of fat tissue can activate neurohormonal systems and promote oxidative stress responses, further exacerbating the cardiovascular burden [37, 38]. The focus of our study was to determine the association between BRI and CVD risk, rather than conducting a detailed investigation of the specific processes involved. Future research should aim to investigate these mechanisms in greater detail, thereby allowing the mechanism by which BRI affects cardiovascular health to be clarified.

## 5. Strengths and Limitations

This study has several strengths. First, it was based on a large-scale, nationally representative sample from the CHARLS, thereby reducing the population heterogeneity and enhancing the generalizability of the results. Second, a longitudinal study design was employed, allowing a more reliable inference of the relationship between BRI and CVD through time-to-event analysis. This distinguishes our study from most previous cross-sectional studies. Furthermore, RCS models revealed a non-linear dose-response relationship between BRI and CVD risk, adding to the novelty of our findings. We also performed subgroup analyses to evaluate the impact of factors such as gender, age, BMI, and comorbidities on the BRI-CVD relationship, allowing us to identify differential effects in various population subgroups and filling gaps in the current literature.

**Fig. 3.**
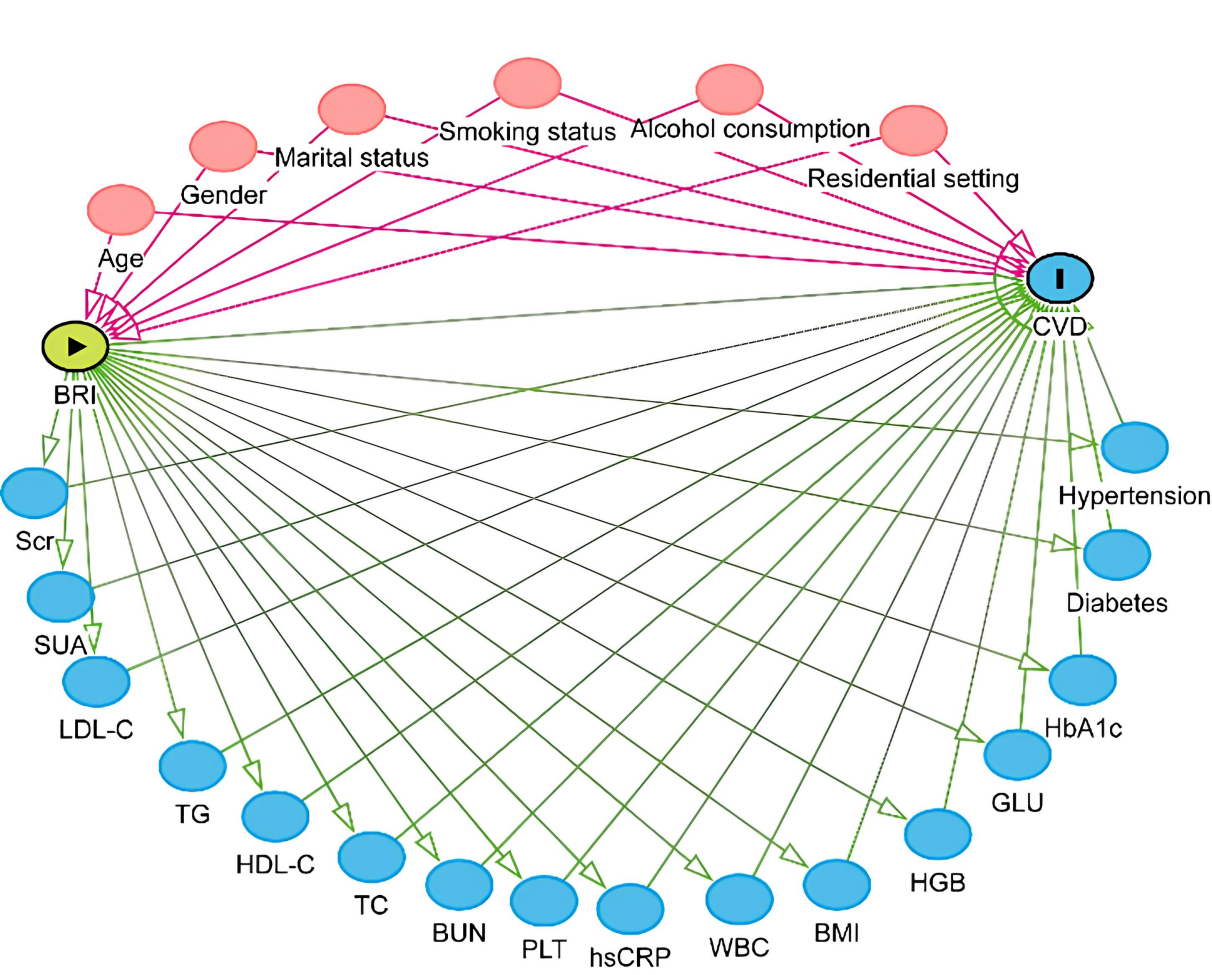
**Directed acyclic graph**.

This study also has several limitations. Although we employed a longitudinal design, causality cannot be definitively established because the study was based on observational data, and hence residual confounding factors may still be present. For instance, unmeasured variables such as dietary habits, physical activity, lifestyle factors, and environmental influences could still potentially affect the risk of CVD. Additionally, while the follow-up period was adequate for preliminary risk assessment, it may be insufficient to fully capture the long-term cardiovascular outcomes. Lastly, BRI was measured only at baseline. Dynamic changes in fat levels over time were not considered and may impact the comprehensive assessment of long-term health effects. Another major limitation is that CVD incidence was determined by physician-diagnosed self-reports. This methodology is susceptible to recall bias and misclassification bias, since certain participants might over-report or under-report their health status due to memory lapses or subjective interpretation. Although the CHARLS dataset is generally reliable, the accuracy of CVD diagnosis is limited by the lack of verification through medical recordings or clinical visits. This potential source of bias should be taken into consideration when interpreting the results of our analysis. Furthermore, the confounding variables included in Model 3 of this study are closely related to the metabolic processes associated with BRI and may act as mediators influencing CVD (Fig. 3). Adjusting for these variables could lead to over-adjustment of the true relationship between BRI and CVD, thereby introducing bias and reducing the interpretability of the model. Future studies should carefully select adjustment variables to avoid over-adjustment and the confounding of mediating effects, ensuring the accuracy of causal inferences.

These findings suggest that future research should have a longer-term, prospective study design. Moreover, it should incorporate repeated BRI measurements and adjustment for other behavioral and environmental variables in order to better elucidate the causality of BRI for CVD.

## 6. Conclusion

In summary, elevated BRI is associated with increased CVD risk. Measurement of BRI may be useful for identifying high-risk individuals and for informing CVD prevention and intervention strategies. Additional research is required to elucidate the specific mechanisms underlying the association between BRI and CVD, therefore allowing better prevention.

## Availability of Data and Materials

The raw data supporting the conclusions of this article are available from the corresponding author upon reasonable request.
